# Radix *Puerariae lobatae* (*Gegen*) suppresses the anticoagulation effect of warfarin: a pharmacokinetic and pharmacodynamics study

**DOI:** 10.1186/s13020-016-0078-9

**Published:** 2016-02-27

**Authors:** Beikang Ge, Zhen Zhang, Zhong Zuo

**Affiliations:** School of Pharmacy, Faculty of Medicine, The Chinese University of Hong Kong, Shatin, New Territories, Hong Kong, China

## Abstract

**Background:**

Radix *Salvia miltiorrhiza* (*Danshen*) and Radix *Puerariae lobatae* (*Gegen*) are used in Traditional Chinese Medicine to treat cardiovascular diseases. However, adverse herb-drug interactions were observed between warfarin and herbal remedies containing *Danshen* and *Gegen*. This study aims to investigate the pharmacokinetic and pharmacodynamic interactions between warfarin and the different components found in *Danshen* and *Gegen*.

**Methods:**

Sixty Sprague–Dawley rats were used to investigate the effects of warfarin (0.2 mg/kg), *Danshen* (240 or 480 mg/kg) and *Gegen* (240 or 480 mg/kg) both in isolation and combination. The rats in the warfarin and *Danshen/Gegen* combination groups were given an oral dose of *Danshen* or *Gegen* 2 h after being given an oral dose of warfarin. After five consecutive days of treatment, the pharmacokinetic interactions between *Danshen/Gegen* and warfarin were investigated by simultaneously monitoring and comparing the cytochrome P450 (CYP) activities, mRNA and protein expression levels in the livers of the rats from the different treatment groups. The pharmacodynamic interactions were evaluated by monitoring and comparing the vitamin K epoxide reductase (VKOR) activities, mRNA and protein expression levels in the livers of rats from the different groups, as well as the thrombomodulin (TM) activities, mRNA and protein in the lungs of these animals. The rat plasma soluble thrombomodulin concentrations of the different treatment groups were also evaluated. Microsomes incubation, Real Time-Polymerase Chain Reaction and Western blot was applied respectively to study the activity, mRNA expression and protein expression of CYP, VKOR and TM.

**Results:**

The activities and expression levels of the CYP and VKOR enzymes in the warfarin-*Gegen* combination groups increased by nearly 30 % (*P* = 0.02) compared with the warfarin-alone group, whereas those of TM decreased by almost 25 % (*P* = 0.02). The administration of *Danshen* did not lead to any changes in the activities or the expression levels of the CYP, VKOR or TM enzymes compared with those of the control group. *Gegen* induced several warfarin-metabolizing CYP enzymes and neutralized the effects of warfarin towards VKOR and TM.

**Conclusion:**

*Gegen*, rather than *Danshen* at the same tested dosage, offsets the anticoagulant effects of warfarin by accelerating the phase I liver metabolism of warfarin, as well as increasing the activity, mRNA and protein expression of VKOR while decreasing those of TM.

**Electronic supplementary material:**

The online version of this article (doi:10.1186/s13020-016-0078-9) contains supplementary material, which is available to authorized users.

## Background

The use of the anticoagulant warfarin in combination with herbal remedies has caused several safety concerns because of the narrow therapeutic window of warfarin [1]. According to reports from pharmacists in the United Kingdom, about 58 % of patients prescribed warfarin also regularly consumed herbal medicines to manage their conditions [2, 3]. A racemic mixture of warfarin consists of a 1:1 mixture of its *R*- and *S*-enantiomers, which differ considerably in terms of their potency and the rate at which they are metabolized [4]. Both of the enantiomers of warfarin are metabolized by cytochrome P450 (CYP) enzymes in a region- and stereo-selective manner, to afford the corresponding 6-, 7-, 8-, 10- and 4′-hydroxywarfarin compounds as the major metabolites [5]. The ingestion of certain plant materials such as grapefruit [6] and garlic [7, 8] can have a pronounced effect on the activity and/or expression of the CYP enzymes, which could have serious consequences for the metabolism of warfarin. The co-administration of herbs and drugs can also affect the activity and expression of vitamin K epoxide reductase (VKOR), which is the target protein of warfarin in terms of its anticoagulant effects. Although there were two separate studies pertaining to the inhibitory activities of the drug-warfarin interactions of paracetamol and sodium dehydroacetate (DHA-S) towards VKOR [9, 10], there were studies concerning the impact of herbs on the activity or expression of VKOR. In addition to VKOR, plasma-soluble thrombomodulin TM (sTM), which is the cleavage product of cellular TM [11], could be used as a potential biomarker for identifying patients at high risk of bleeding during warfarin treatment [12–14]. Notably, Salvianolic acid B led to an increase in the activity and expression of TM in vitro [15]. *Danshen*-*Gegen* formula (DGF), which is a herbal medicine containing Radix *Salvia miltiorrhiza* (*Danshen*) and Radix *Puerariae lobatae* (*Gegen*), led to a 5.36-fold increase in the mRNA expression of TM [16].

DGF, which is the aqueous extract derived of a 7:3 (w/w) mixture of *Danshen* and *Gegen*, was recently developed in Hong Kong [17]. DGF contains a variety of active components, including danshensu, salvianolic acid B (SAB), protocatechuic aldehyde (PCA), puerarin, daidzin and daidzein [18], and was reported to be an effective agent for the treatment of cardiovascular diseases [19–21]. The results of our previous in vivo study in rats demonstrated that the co-administration of DGF with warfarin led to pronounced herb-drug interactions [22], including a significant decrease in the C_max_, area under curve (AUC) and the prothrombin (PT) time of warfarin. Although these changes in the pharmacokinetic properties of warfarin were attributed to the DFG-mediated induction of the CYP enzymes, the precise nature of the herbal components responsible for these induction effects were identified.

*Danshen* was reported to improve microcirculation, cause coronary vasodilation, suppress the formation of the thromboxane and inhibit the adhesion and aggregation of platelets. Based on its many interesting biological properties, *Danshen* has been widely used to treat coronary artery disease and numerous other cardiovascular diseases in China, Korea and Japan, as well as several other Asian countries [23–25]. Although the results of several previous studies suggested that *Danshen* interacts with warfarin by changing its pharmacokinetic and pharmacodynamic parameters [26–29], these studies generally fail to provide dosage information for *Danshen.* Furthermore, there are several inconsistencies in the findings of these studies. Preclinical studies in this area not reached an agreement on the effects of *Danshen* on the PT time of warfarin after the co-administration of these agents [30, 31]. The discrepancies observed in these pre-clinical and clinical research data could be explained in terms of the differences in the administration routes, dosages and *Danshen* preparations used in the different studies. In this study, we used an ex vivo approach to mimic the clinical behaviors of warfarin and *Danshen* and investigate the underlying mechanism of interaction between these two agents. Compared with *Danshen*, there were very few reports pertaining to the herb-drug interactions of *Gegen*, which is another major component of DGF. In a similar manner to *Danshen*, *Gegen* was reported to improve micro-circulation, increasing blood flow and preventing coronary artery disease [32]. Puerarin and *Gegen* extract were reported to regulate the activity of certain CYP enzymes in rats [33]. Furthermore, the results of a recent clinical study showed that puerarin induced the activity of CYP1A2 and inhibited the activity of CYP2D6 [34], which indicated that there could be potential interactions between warfarin and *Gegen*.

Existing mechanistic studies mainly focused on the impact of changes in the pharmacokinetics of warfarin rather than its pharmacodynamics, despite the fact that changes in the latter account for 79.9 % of all the identifiable herb-drug interaction mechanisms [35]. This study aims to investigate the pharmacokinetic and pharmacodynamic interactions between warfarin and *Danshen*/*Gegen*. To achieve these goals, we applied an ex vivo approach to investigate the effects of *Danshen/Gegen* on the different CYP enzymes involved in the metabolism of warfarin (for pharmacokinetic analysis). We also monitored the effects of *Danshen/Gegen* on the activities and mRNA and protein expression levels of VKOR and TM (for pharmacodynamic analysis).

## Methods

### Herbs

The concentrated Chinese medicine granules of *Danshen* (Batch No. 1007) and *Gegen* (Batch No. 1027) with the extraction ratio of 1:5 were purchased from Nong’s Company Limited (Hong Kong). The raw herbs were morphologically authenticated by an in-house herbalist and chemically by the thin layer chromatography in accordance with the Chinese Pharmacopoeia 2005 [36]. The plant names in current study were used according to the latest revision in “The Plant List” (http://www.theplantlist.org).

### Reagents

Racemic warfarin, diclofenac sodium and naringin (internal standards) were purchased from Sigma-Aldrich (St. Louis, MO, USA). The standards of sodium danshensu, protocatechuic aldehyde, SAB, puerarin, daidzin and daidzein were purchased from Zhongxin Innova Laboratories (Tianjin, China). Phenobarbital (PB), beta-naphthoflavone (BNF), cyclophosphamide monohydrate (CPA), bovine serum albumin, S-2238 and human thrombin were purchased from Sigma-Aldrich (St. Louis, MO, USA). Vitamin K_1_ purchased from Sigma was used to prepare vitamin K_1_ 2, 3-epoxide (VKO) by the method of Tishler [37]. Hirudin was purchased from Calbiochem (La Jolla, California, USA). Acetic acid and ethyl acetate were obtained from BDH Laboratory (Poole, England). Acetonitrile and methanol (HPLC grade) were obtained from Tedia Company Inc. (Fairfield, USA) and Fisher Scientific UK Limited (Leicestershire, UK), respectively. All other reagents were analytical or HPLC grade. Distilled and deionized water was used for the preparation of all solutions. *S*-warfarin, *R*-warfarin, and racemic standards of 4′-, 6-, 7-, 8- and 10-OH-warfarin were purchased from Ultrafine Chemicals (Manchester, UK). Both the primary and secondary antibodies of CYP1A1, CYP2B1/2B2, CYP2C6, CYP2C11, VKOR, TM and GAPDH were purchased from Abcam (Abcam Inc., Cambridge, MA). ELISA kits for detecting sTM plasma concentration were purchased from Life Science Inc.

### Animals

Healthy male SD rats (220–250 g) were supplied by the Animal Service Center at the Chinese University of Hong Kong and fasted overnight before use. The experiments were carried out under the approval by the Animal Ethics Committee of the Chinese University of Hong Kong with an approval number of (12-538) in DH/HA&P/8/2/1 Pt.26 (Additional file 1).

### Instruments for sample analyses

The LC/MS/MS system, used for sample analyses of warfarin and its monohydroxylated metabolites, were performed with an ABI 2000 tandem triple quadruple LC/MS spectrometer equipped with an electrospray ionization source (ESI), two Perkin-Elmer PE-200 series micro-pumps and auto-sampler (Perkin-Elmer, Norwalk, CT, USA). The liquid chromatographic sample analyses instrument for detecting VK and VKO was a Waters HPLC system (Waters, Milford, MA,USA) equipped with 996 photodiode-array detector and 2695 separation module (pump and auto sampler). The chromatographic separation was performed at ambient temperature by X-Bridge™ C_18_ column (150 × 4.6 mm i.d., 3.5 µm particle size). The auto-sampler was set at 4 °C. Acetonitrile/isopropanol/water in a ratio of 100/8/2 (*v*/*v*/*v*) was used as mobile phase, with the flow rate of 1.2 mL/min. Absorbance of the eluting materials was measured at 250 nm. The Bio-Rad microplate reader from BioRad (Benchmark, Japan) was used to analyze the optical destiny of samples from ELISA and TM activity tests.

### Standardization of Danshen and Gegen granules

Identification and quantification of the major components in *Danshen* and *Gegen* granules were performed by Waters HPLC according to our previous study with modifications [22]. The chromatographic separation of the analytes was achieved by an Agilent Eclipse XDB-C18 column (250 × 4.6 mm i.d., 5 μm particle size). The mobile phase consisting of 0.5 % acetic acid in acetonitrile (solvent A) and 0.5 % acetic acid in water (solvent B) was run with gradient elution at a flow rate of 1 mL/min. The linear gradient elution was carried out as follows with a total running time of 90 min: solvent A was kept at 5 % for the first 5 min, and then increased to 10, 17, 35 and 90 % in the next 13, 12, 10 and 30 min, respectively, then returned to 5 % in 5 min and equilibrated for 15 min before the next injection. HPLC analyses indicated that the marker compounds (µg/100 mg granules, mean ± SD, n = 3) for *Danshen* granules were danshensu (1140.3 ± 24.6), SAB (821.3 ± 33.1), PCA (82.2 ± 4.6), and that for *Gegen* granules were puerarin (1314.9 ± 44.1), daidzein (74.4 ± 9.4) and daidzin (89.2 ± 7.2), respectively.

### Animal treatments

The dosage of *Danshen* and *Gegen* for rats were both 240 mg/kg/day (twice a day), which were calculated from its respective clinical therapeutic dose recommended by Chinese Pharmacopeia [36]. Since the clinical therapeutic dosage of warfarin in common use was 1–2 mg/day for a person, the equivalent rats dosage was calculated to be 0.2 mg/kg (human equivalent dosage in rat = human dosage/60 kg × 6.2), once daily [38]. Healthy male SD rats were divided into nine groups (n = 6) and each group was treated for five consecutive days with different substance, including *Danshen* single dose (*Danshen*×1: 240 mg/kg/day), *Danshen* double dose (*Danshen*×2: 480 mg/kg/day), warfarin plus *Danshen* single dose (War + *Danshen*×1: 0.2 + 240 mg/kg/day), warfarin plus *Danshen* double dose (War + *Danshen*×2: 0.2 + 480 mg/kg/day), *Gegen* single dose (*Gegen*×1: 240 mg/kg/day), *Gegen* double dose (*Gegen*×2: 480 mg/kg/day), warfarin plus *Gegen* single dose (War + *Gegen*×1: 0.2 + 240 mg/kg/day), warfarin plus *Gegen* double dose (War + *Gegen*×2: 0.2 + 480 mg/kg/day) and warfarin alone (War: 0.2 mg/kg/day). *Danshen/Gegen* was given to rats twice daily. In the combination groups, Danshen/Gegen was given to rats 2 h after the oral dosing of warfarin. In addition to the drug-treated groups, H_2_O, phenobarbital (PB 60 mg/kg/day), beta-naphthoflavone (BNF 40 mg/kg/day) and cyclophosphamide (CPA 40 mg/kg/day) were also given orally for five consecutive days to rats that formed into one vehicle control and three positive controls (for determining the activity and expressions of CYPs) respectively. Specifically, BNF for CYP1A1, PB for CYP2B1 and CYP2C6 and CPA for CYP2C11, were used to validate the assay. For the determining the activity and expressions of VKOR and TM, warfarin was regarded as the positive control for the assay validation. Two hours after the last dosing, rats were sacrificed by decapitation. Livers, lungs and plasma were obtained from each rat for further treatment and assay.

### Tissue sample preparations

#### Rat liver microsome (RLM) preparation

The rat livers were perfused by saline before being excided surgically on ice and cut into pieces for storage at −80 °C. The liver microsomes were prepared by series centrifugation as described previously [39]. Briefly, liver samples were thawed and weighed before homogenization medium were added. The tissue was chopped using scissors and homogenized with an automatic homogenizer at 500 rpm (IKA Labortechnik, Staufen, Germany). The resultant homogenates were transferred to clean centrifuge tubes, and centrifuged (Beckman Colter, Inc, Fullerton, CA, USA) at 20,000×*g* for 20 min at 4 °C. The supernatant was collected and further centrifuged at 100,000×*g* for 1 h under 4 °C by the L8-70 Beckman ultracentrifuge. The obtained microsomal pellet was re-suspended with homogenization medium and stored at −80 °C. Protein concentrations of the prepared liver microsomes were determined by the Bio-Rad protein assay kit (Bio-Rad Pacific Ltd, Hong Kong) and analyzed by the micro-plate reader (Benchmark, Japan) [40].

#### Extraction of TM from rat lungs

Rat lungs were perfused by saline before being excided surgically on ice and cut into pieces for storage at −80 °C. Pieces of lung were then homogenized in a glass tissue grinder of 50 mL at 4 °C. Centrifuge was performed at 30,000×*g* for 40 min at 4 °C to get the pellet, which was re-suspended in 250 mL buffer (0.25 M sucrose, 0.02 M Tris–HCl and 1 mM benzamidine-HCl, pH 7.5), homogenized, and centrifuged again as described above. The TM was finally extracted from the pellet by homogenization in 0.25 M sucrose, 0.02 M Tris–HCl, 1 mM benzamidine-HCl, and 0.5 % (*v*/*v*) Triton X-100, pH 7.5. This process was repeated three times and TM activity was assessed in the resulting extract [41]. The protein concentration of the extract was measured with Bio-Rad protein assay kit and adjusted to 100 ng/mL.

## Methods for pharmacokinetic studies

### CYP activities, mRNA and protein expressions in rat liver

#### Rat liver microsomal activities for mono-hydroxylation of warfarin

The activities of rat liver microsomes for metabolizing warfarin were detected according to methods from our previous study [22]. A typical incubation (100 μL) contained 50 mM Tris–HCl buffer (pH 7.4), 10 mM MgCl_2_, 1.0 mg/mL microsomal protein, and racemic-, *R*- or *S*-warfarin as substrate respectively (5 μM for *R*- and *S*-warfarin, 10 μM for racemic warfarin). The mixture was pre-warmed in a 37 °C water bath with gentle shaking for 10 min before addition of NADPH (final concentration of 1.0 mM) into the mixture to initiate the reaction. About 0.1 mL ice-chilled acetonitrile containing internal standard (2.0 μg/mL of diclofenac sodium) was added and incubated for another 30 min. After the mixture centrifugation, the supernatant was subjected to LC/MS/MS to compare the mono-hydroxywarfarin metabolites (4′-, 6-, 7-, 8- and 10-hydroxywarfarin) among different treatment groups. The activities of CYPs could be expressed as the amount of mono-hydroxywarfarin metabolites formed.

#### CYP mRNA expression

Approximately 30 mg of rat livers were homogenized, and RNA was isolated with the Qiagen RNA extraction kit (Valencia, CA, USA). The quality of RNA was evaluated by measuring the 260/280 ratio (˃1.9) with an ultraviolet light spectrophotometer (Shimadzu, Japan). The sequence of the sense and antisense primers for rat CYP1A1 (FP: CCAAACGAGTTCCGGCCT; RP: TGCCCAAACCAAAGAGAATGA) [42], CYP2B1 (FP:AACCCTTGATGACCGCAGTAAA; RP: TGTGGTACTCCAATAGGGACAAGATC) [42], CYP2C6 (FP: ATAAGACGCTTTACCCTCAC; RP: GATTTTCCTGCTTCCACTTA) [43], CYP2C11 (FP: TGCCCCCTTTTTACGAGGCT; RP: GGAACAGATGACTCTGAATTCT) [44] and GAPDH (FP: CAAGGTCATCCATGACAACTTTG; RP: GGGCCATCCACAGTCTTCTG) [42] gene were designed as described previously. The reaction mixture system (final volume 20 µL) was prepared as follow: 4 units multiscribe reverse transcriptase, 1×RT buffer, 1 mM random hexamer primers, 0.5 mM each of dATP, dGTP, dCTP and dTTP, and 10 mL total mRNA extract. The PCR involved an initiating heating for 10 min at 95 °C, 40 cycles consisting of 10 s at 95 °C, 20 s at 60 °C, and a final extension step of 30 s at 72 °C. The fold change of target gene expression level in different drug treatment groups (after correction with the level of GAPDH) was determined by the following equation: Fold change = 2^−Δ(ΔCt)^, where ΔCt = Ct(target)−Ct(GAPDH) and Δ(ΔCt) = ΔCt(treated)−ΔCt(vehicle control) [22]. The Ct (cycle threshold) in the equation was defined as the number of cycles required for the fluorescent signal to cross the threshold.

#### CYP protein expression

The levels of CYP1A1, 2B1/2, 2C6, 2C11 and GAPDH proteins in rat liver microsomes obtained from different treatment groups were determined by Western blotting. Sodium dodecyl sulfate (SDS)–PAGE was performed by a Mini-Protean II system (Bio-Rad) as described previously [45]. After denaturing with sample buffer, rat liver microsomal proteins were separated in a 10 % (*w*/*v*) SDS–polyacrylamide gel and blotted onto a PVDF membrane (Immobilon transfer membrane, Millipore, Billerica, MA, USA). The membrane was blocked in 5 % defatted milk which was then incubated with the primary and secondary antibodies (horseradish peroxidase labeled anti-mouse or anti-rabbit IgG). The membrane was then immersed in the enhanced chemiluminescence solution (Millipore, Billerica, MA, USA) for 60 s. Digital chemiluminescence images were captured and analyzed by FluorChem Q Imaging System (Alpha Innotech Corporation, Santa Clara, CA, USA).

## Methods for pharmacodynamic studies

### VKOR activity, mRNA and protein expression in rat livers

#### VKOR activity

Rat liver microsomes of different treatment groups were rinsed and re-suspended in the 0.02 M Tris–HCl buffer (pH 7.4) to obtain a microsomal protein concentration of 5 mg/mL 0.02 mL of this microsomal suspension was mixed with 0.071 mL of the 0.02 M Tris–HCl buffered solution (pH 7.4). The mixture was pre-incubated for 3 min at 30 °C. Tris–HCl buffered solution (pH 7.4, 2 µL 0.02 M) containing 0.1 M DTT (Sigma-Aldrich, St. Louis, MO, USA) were added, mixed and incubated for 5 min at 30 °C. Synthesized VKO (10 nM) in 2 µL of isopropanol was also added and subsequently incubated for 60 min at 30 °C. The reaction was stopped by the addition of 0.1 mL of ice-chilled isopropanol. Tocopheryl acetate (2.5 µg; Sigma-Aldrich, St. Louis, MO, USA) in 0.2 mL of hexane was added as the internal standard. After vortex for 1 min, the mixture was centrifuged at 15,000×*g* for 5 min. The hexane phase was evaporated to dryness with nitrogen [39]. The residue was dissolved in 150 µL mixed buffer containing acetonitrile/isopropanol/water (100/8/2: *v*/*v*/*v*), and 10 µL was analyzed by HPLC. The amount of vitamin K_1_ formed was determined from the peak height ratio between vitamin K_1_/internal standard and a calibration graph. VKOR activity was expressed as the amount of vitamin K_1_ formed.

#### VKOR mRNA and protein expression

The sequence of the sense and antisense primers for rat VKOR gene (VKORC1) used in RT-PCR was designed as described previously [46]. RT-PCR procedures for detecting mRNA expression of VKORC1 were same as that described for CYP mRNA expression. VKOR protein expression detection was conducted with the same operations for CYP protein expression.

### TM activity, mRNA and protein expression in rat lung

#### TM activity

The ability of TM to accelerate protein C activation by thrombin was tested by a chromogenic assay as follow: 0.5 M of purified protein C (Stago) in 100 µL of buffer was activated by 10 nM (1 NIH unit) of human thrombin (Sigma-Aldrich, St. Louis, MO, USA) in 10 µL of buffer in the presence of 5 mM calcium, 3 mg/mL of bovine serum albumin and of 50 µL of lung extracts from different substance treated groups. The final volume was 200 µL. Incubation of the mixture lasted 30 min at 37 °C. The activation phase was stopped by adding 2 units of hirudin. The hydrolytic activity of the activated protein C (APC) thus generated was assessed by spectrophotometric measurement at 405 nm of the rate of hydrolysis of D-Phe-Pip-Arg-pNA·2HCl, that is, S-2238. For this purpose, 800 µL of Tris–HCl buffer, pH 7.5, containing 0.1 mM of S-2238 was added to the mixture (final volume: 1 mL). The changes in optical density per minute were converted into units of APC formed by referring to a standard curve constructed with concentrations of 20, 40, 80, 160 and 320 n of standard APC (Stago). The activity of TM could be expressed as the amount of APC formed during incubation [47].

#### TM mRNA and protein expression

TM mRNA and protein (extracted from rat lung) expression were detected as that described for CYP. The sequence of the sense and antisense primers for rat TM used in RT-PCR was designed as described previously [48].

### sTM plasma concentration analysis

After the animal treatment described previously, blood was collected using 3.8 % sodium citrate (sodium citrate: blood = 1:9) as an anticoagulant. Plasma was obtained by centrifuging the blood at 15,000×*g* for 3 min. The plasma samples were frozen and stored at −80 °C until the assay. An enzyme-linked immunosorbent assay (ELISA) was used to measure the plasma concentration of sTM. Standard sTM was series diluted to make a calibration curve. The concentration of sTM was determined from the optical density of each sample at 450 nm against a calibration graph.

### Statistical analysis

Data were expressed as the mean ± SD. Differences between two groups were analyzed by an unpaired student’s *t* test. Differences between groups for continuous variables were evaluated by one way ANOVA with post hoc Tukey (when variances were equal according to Levene’s test) or Games-Howell test (when variances were not equal according to Levene’s test) [49]. *P* value less than 0.05 was considered as statistically significant.

## Results

### Pharmacokinetic mechanisms of warfarin-*Gegen* interaction

#### Gegen *induced the activities and mRNA expressions of different CYPs in rat liver*

##### Induction on CYP activities for warfarin metabolism

**Fig. 1:**
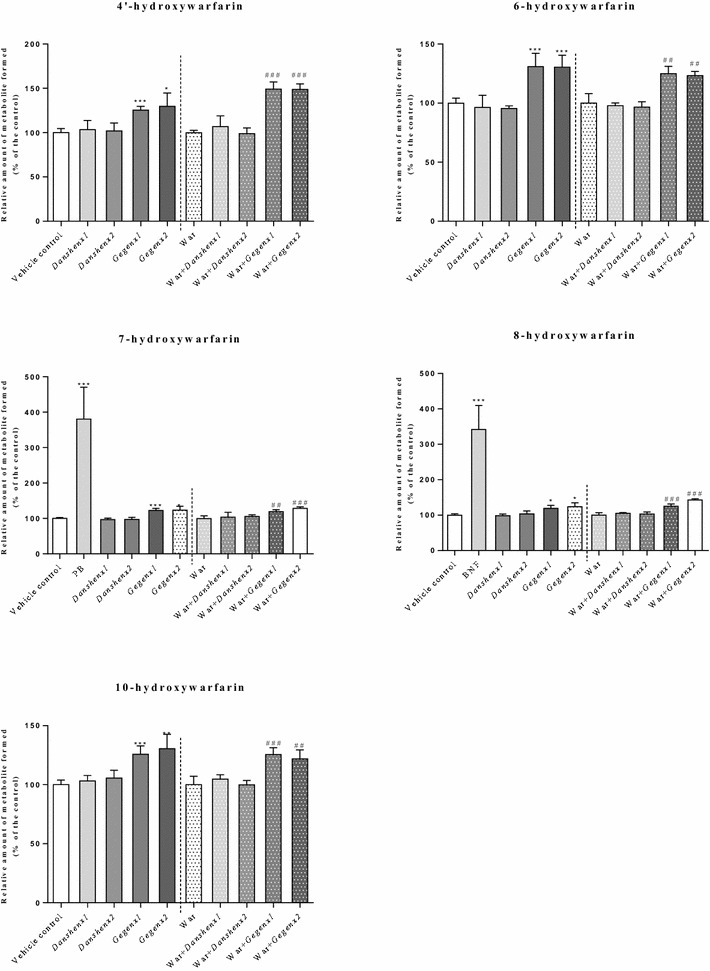
Relative amount of formed monohydroxywarfarin (% of control) from different treatment groups. Using *R*-warfarin as substrate. *P < 0.05; **P < 0.01; ***P < 0.001, compared with the vehicle control group. ^#^ *P* < 0.05; ^##^ *P* < 0.01; ^###^ *P* < 0.001, compared with the warfarin alone group. ^&^ *P* < 0.05; ^&&^ *P* < 0.01; ^&&&^ *P* < 0.001, compared within same groups of different doses. Each point represents the mean ± SD (n = 6)

When *R*-warfarin was used as the substrate (5 μM), the formation of all five mono-hydroxylated warfarin metabolites in the *Gegen*×1 and *Gegen*×2 groups increased significantly by 19–31 % (*P* = 0.03) and 24–30 % (*P* = 0.03), respectively, relative to the vehicle control group. Compared with the combined amount of all five mono-hydroxylated warfarin metabolites formed in the warfarin-alone group, there were significant increases in both of the combination groups (i.e., War + *Gegen*×1 and War + *Gegen*×2) of 19–49 % (*P* = 0.02) and 22–49 % (*P* = 0.03), respectively. No significant differences were observed in the amounts of the mono-hydroxylated metabolites formed between the vehicle control and *Danshen* groups. In the positive control groups, the formation of 7- and 8-hydroxylated warfarin increased by 281 % (*P* < 0.001) and 242 % (*P* < 0.001) when the animals were treated with PB and BNF (positive controls for the activities of rat CYP2C6 and CYP1A1, respectively, contributing to the formation of 7- and 8-hydroxylated warfarin) [22], respectively, compared with the results of the vehicle control group (Fig. 1).

**Fig. 2:**
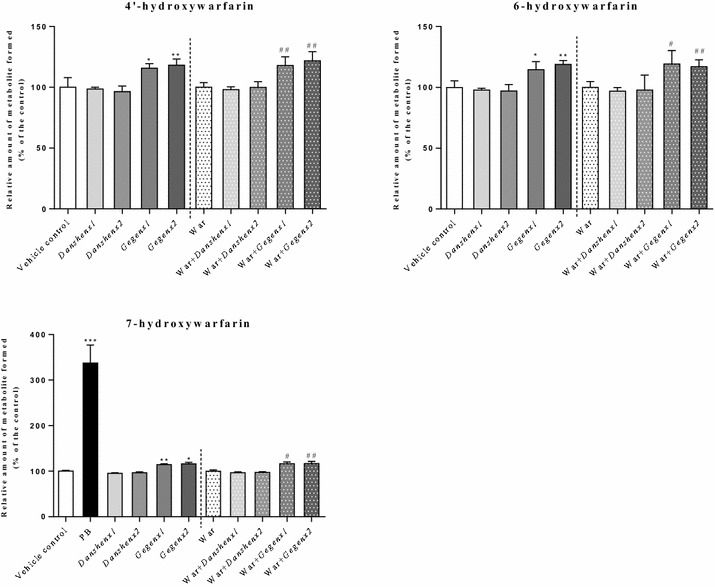
Relative amount of formed monohydroxywarfarin (% of control) from different treatment groups. Using S-warfarin as substrate. *P < 0.05; **P < 0.01; ***P < 0.001, compared with the vehicle control group. ^#^ *P* < 0.05; ^##^ *P* < 0.01; ^###^ *P* < 0.001, compared with the warfarin alone group. ^&^ *P* < 0.05; ^&&^ *P* < 0.01; ^&&&^ *P* < 0.001, compared within same groups of different doses. Each point represents the mean ± SD (n = 6)

When *S*-warfarin was used as a substrate (5 μM), the formation of the three detectable mono-hydroxylated warfarin metabolites (i.e., 4′-, 6- and 7-hydroxylated warfarin) increased significantly by 14–16 % (*P* = 0.04) and 16–19 % (*P* = 0.03) in the *Gegen*×1 and *Gegen*×2 groups, respectively, compared with the vehicle control group. Compared with the combined amount of the 4′-, 6- and 7-monohydroxylated warfarin metabolites formed in the warfarin-alone group, we observed significant increases of 17–19 % (*P* = 0.04) and 17–22 % (*P* = 0.03) in the formation of these metabolites in the *Gegen*×1 and *Gegen*×2 groups, respectively. No inductive effects were observed in the *Danshen*-treated groups based on a comparison of the warfarin metabolites formed in this group with those in the vehicle control group. In the positive control groups, the formation of 7-hydroxywarfarin increased by 237 % (*P* < 0.001) when the rats were treated with PB (positive controls for rat CYP2C6, contributing to the formation of 7-hydroxywarfarin) compared with the vehicle control group (Fig. 2).

**Fig. 3:**
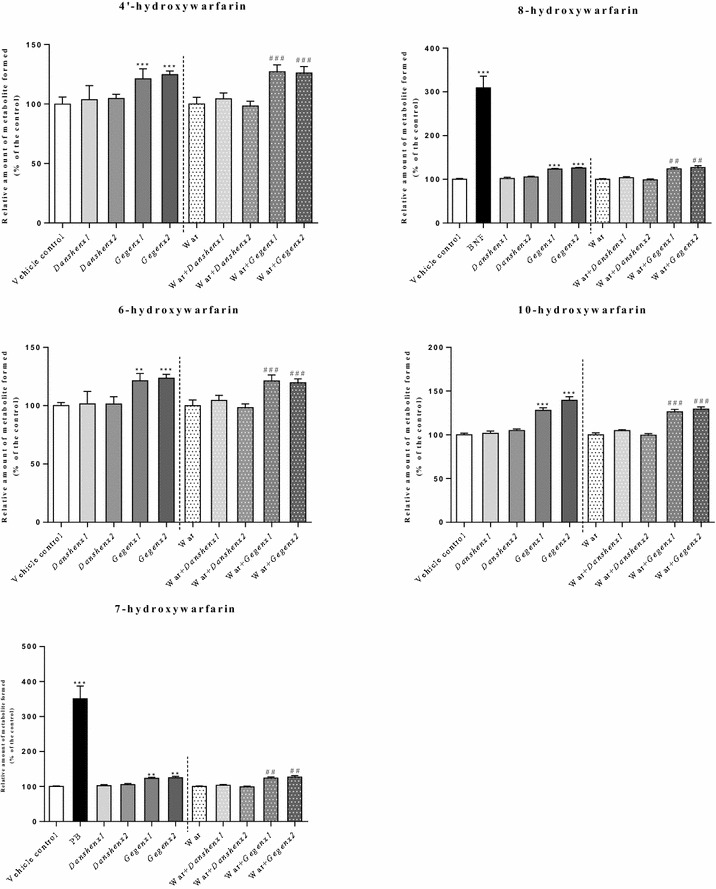
Relative amount of formed monohydroxywarfarin (% of control) from different treatment groups. Using racemic warfarin as substrate. *P < 0.05; **P < 0.01; ***P < 0.001, compared with the vehicle control group. ^#^ *P* < 0.05; ^##^ *P* < 0.01; ^###^ *P* < 0.001, compared with the warfarin alone group. ^&^ *P* < 0.05; ^&&^ *P* < 0.01; ^&&&^ *P* < 0.001, compared within same groups of different doses. Each point represents the mean ± SD (n = 6)

When racemic warfarin was used as the substrate (10 μM containing 5 μM of the *R*- and *S*-enantiomers) there were significant increases of 21–28 % (*P* = 0.03) and 24–39 % (*P* = 0.02) in the formation of the 4′-, 6-, 7-, 8- and 10-hydroxywarfarin metabolites in the *Gegen*×1 and *Gegen*×2 groups, respectively, compared with the vehicle control group. Compared with the warfarin-alone group, the co-administration of *Gegen*×1 and *Gegen*×2 with warfarin led to significant increases in the formation of all five mono-hydroxylated warfarin metabolites by 21–27 % (*P* = 0.03) and 20–29 % (*P* = 0.03), respectively. No inductive effect was observed when for the warfarin metabolites formed in the *Danshen*-treated groups based on a comparison of these results with those of the vehicle control group. In the positive control groups, the formation of the 7- and 8-hydroxywarfarin metabolites increased by 251 % (*P* < 0.001) and 209 % (*P* < 0.001) when the rats were treated with PB and BNF, respectively, compared with the vehicle control group (Fig. 3).

##### Induction of the mRNA expression of CYP1A1, CYP2B1, CYP2C6 and CYP2C11

**Fig. 4:**
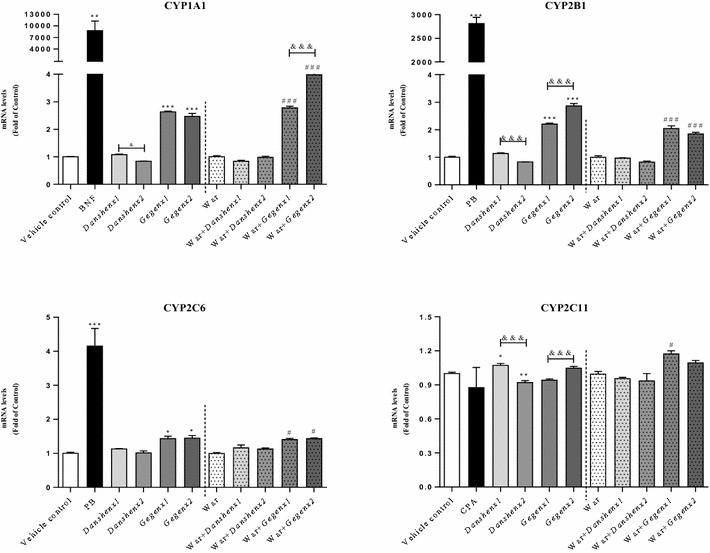
Rat liver mRNA expression (Fold of Control) for target CYP isoforms from different treatment groups. *P < 0.05; **P < 0.01; ***P < 0.001, compared with the vehicle control group. ^#^ *P* < 0.05; ^##^ *P* < 0.01; ^###^ *P* < 0.001, compared with the warfarin alone group. ^&^ *P* < 0.05; ^&&^ *P* < 0.01; ^&&&^ *P* < 0.001, compared within same groups of different doses. Each point represents the mean ± SD (n = 6)

Compared with the vehicle control group, there were significant increases of 260 % (*P* < 0.001) and 220 % (*P* < 0.001) in the mRNA expression levels of CYP1A1 and CYP2B1 in the *Gegen*×1 group, respectively. Similar increases of 250 % (*P* < 0.001) and 290 % (*P* < 0.001) were also observed in the *Gegen*×2 group, respectively (Fig. 4). However, these increases were small in size compared with those of the positive control groups. For example, BNF afforded a 8761-fold increase, whereas PB gave a 2812-fold increase. Compared with the mRNA expression ratio of CYP1A1 to CYP2B1 in the warfarin-alone group, there were significant increases (*P* < 0.001) in the counterpart ratios of both combination groups (i.e., War + *Gegen*×1 and War + *Gegen*×2). For example, the former of these two groups (i.e., War + *Gegen*×1) showed 2.8- and 2.1-fold increases for CYP1A1 and CYP2B1, respectively, whereas the latter group (i.e., War + *Gegen*×2) showed 4.0- and 1.9-fold increases for CYP1A1 and CYP2B1, respectively. No inductive effects were observed in the mRNA expression levels of CYP1A1 and CYP2B1 when the *Danshen*-treated group was compared with the vehicle control group.

PB was used as a positive control to detect the mRNA expression of CYP2C6. Compared with the vehicle control group, there was a significant increase of 420 % (*P* < 0.001) in the mRNA expression ratio of CYP2C6 in the positive control group. Furthermore, the mRNA expression levels of CYP2C6 increased significantly by 1.4- and 1.5-fold in the *Gegen*×1 (*P* = 0.03) and *Gegen*×2 (*P* = 0.02) groups, respectively, compared with the vehicle control group. A significant 1.4-fold (*P* = 0.03) increase in the mRNA expression level of CYP2C6 was also observed in both of the *Gegen* combination groups (i.e., War + *Gegen*×1 and War + *Gegen*×2), compared with the warfarin-alone group. No significant differences were observed in the CYP2C6 mRNA expression ratios between the *Gegen*×1 and *Gegen*×2 groups. Furthermore, no inductive effects were observed in the mRNA expression level of CYP2C6 for both doses of *Danshen* compared with the vehicle control group.

In terms of the mRNA expression of CYP2C11, we observed a slight increase of 1.07-fold in the *Danshen*×1 group (*P* = 0.04) compared with the vehicle control group. A small increase of 117 % (*P* = 0.02) was also observed in the mRNA expression of CYP2C11 in the War + *Gegen*×1 group compared to the warfarin-alone group.

##### No significant effects on the rat liver CYP protein expressions

**Fig. 5:**
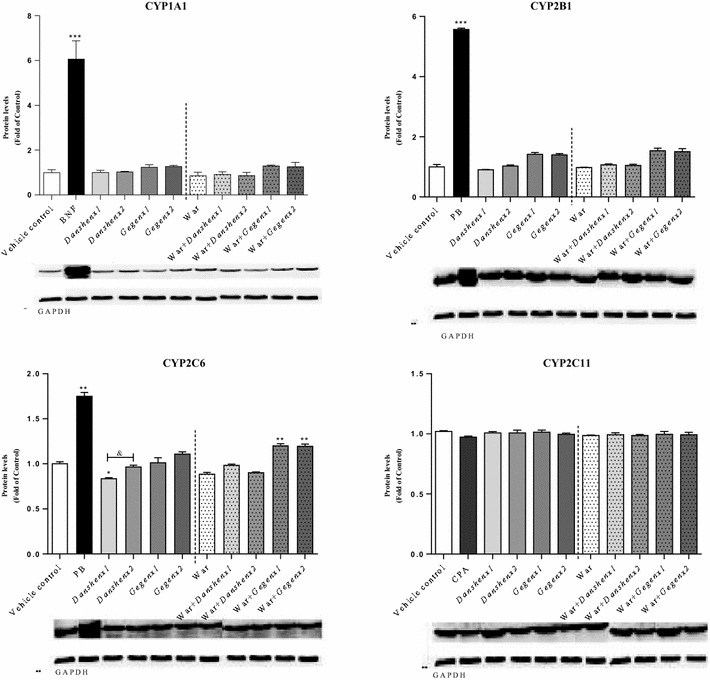
Protein level (Fold of Control) for target rat liver CYP isoforms from different treatment groups. *P < 0.05; **P < 0.01; ***P < 0.001, compared with the vehicle control group. ^#^ *P* < 0.05; ^##^ *P* < 0.01; ^###^ *P* < 0.001, compared with the warfarin alone group. ^&^ *P* < 0.05; ^&&^ *P* < 0.01; ^&&&^ *P* < 0.001, compared within same groups of different doses. Each point represents the mean ± SD (n = 6)

Changes in the protein expression levels of the target CYP isozymes were determined to be minor compared with those observed for the metabolic activity and mRNA expression levels (Fig. 5). In the *Gegen*×1 and *Gegen*×2 groups, the expression level of CYP2C6 protein appeared to increase compared with the vehicle control group, but this increase was not statistically significant. No inductive effects were observed in the expression levels of any of the target CYP proteins in the *Danshen*-treated groups compared with the vehicle control group. In the positive control groups, the expression levels of the CYP1A1, CYP2B1/2B2 and CYP2C6 proteins increased significantly by 6.1- (*P* < 0.001), 5.6- (*P* < 0.001) and 1.7-fold (*P* = 0.003), respectively, compared with the vehicle control group.

## Pharmacodynamic mechanisms of the warfarin-*Gegen* interaction

### Gegen led to an increase in the activity, as well as the mRNA and protein expression levels of VKOR in rat liver

#### Induction on VKOR activity

**Fig. 6:**
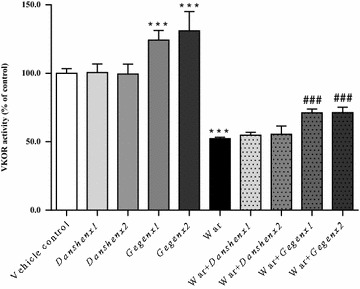
Fold of change in VKOR activity from different treatment groups. *P < 0.05; **P < 0.01; ***P < 0.001, compared with the vehicle control group. ^#^ *P* < 0.05; ^##^ *P* < 0.01; ^###^ *P* < 0.001, compared with the warfarin alone group. ^&^ *P* < 0.05; ^&&^ *P* < 0.01; ^&&&^ *P* < 0.001, compared within same groups of different doses. Each point represents the mean ± SD (n = 6)

Compared with the vehicle control group, the *Danshen*×1 and *Danshen*×2 groups showed no significant changes in their VKOR activity. In contrast, there were significant increases of 124.4 ± 6.3 % (*P* < 0.001) and 131.1 ± 12.9 % (*P* < 0.001) in the VKOR activities of the *Gegen*×1 and *Gegen*×2 groups, respectively. Warfarin reduced the activity of VKOR by 52.4 ± 5.1 % (*P* < 0.001) compared with the vehicle control group, which was consistent with its therapeutic effect. A comparison of the warfarin-alone group with the four different combination groups (i.e., War + *Danshen*×1; War + *Danshen*×2; War + *Gegen*×1; War + *Gegen*×2) revealed that *Danshen* had no impact on the VKOR activity during warfarin treatment. In contrast, the co-administration of *Gegen* with warfarin led to significant increases of 35.9 ± 4.9 % (*P* < 0.001) and 36.2 ± 6.9 % (*P* < 0.001) in the VKOR activities of the War + *Gegen*×1 and War + *Gegen*×2 groups, respectively. However, it was not possible to completely offset the inhibitory activity of warfarin towards VKOR using *Gegen* (Fig. 6).

#### Up-regulation in the mRNA and protein expression levels of VKOR

**Fig. 7:**
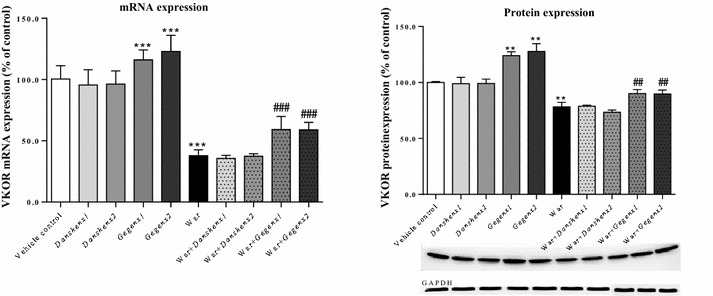
Fold of change in VKOR mRNA (*left*) and protein (*right*) expression from different treatment groups. *P < 0.05; **P < 0.01; ***P < 0.001, compared with the vehicle control group. ^#^ *P* < 0.05; ^##^ *P* < 0.01; ^###^ *P* < 0.001, compared with the warfarin alone group. ^&^ *P* < 0.05; ^&&^ *P* < 0.01; ^&&&^ *P* < 0.001, compared within same groups of different doses. Each point represents the mean ± SD (n = 6)

Compared with the vehicle control group, there were no significant changes in the mRNA and protein expression levels of VKOR in the *Danshen*×1 and *Danshen*×2 groups. In contrast, there were significant increases of 116.1 ± 6.6 % (*P* < 0.001) and 123.0 ± 10.8 % (*P* < 0.001) in the mRNA expression of VKOR in the *Gegen*×1 and *Gegen*×2 groups, respectively, compared with the vehicle control group. Significant increases of 123.9 ± 2.9 % (*P* = 0.002) and 127.8 ± 5.7 % (*P* = 0.002) were also observed in the expression levels of the VKOR protein in the *Gegen*×1 and *Gegen*×2 groups, respectively, compared with the vehicle control group. Warfarin led to significant reductions of 43.7 ± 13.9 % (*P* < 0.001) and 71.6 ± 11.4 % (*P* = 0.004) in the mRNA and protein expression levels of VKOR, respectively compared with the vehicle control group. Compared with the mRNA and protein expression levels of VKOR in the warfarin-alone group, there were significant increases (*P* < 0.001) of 27 % (mRNA expression) and 25 % (protein expression) in the corresponding *Gegen* combination groups, respectively. However, the co-administration of *Danshen* with warfarin had no discernible impact of the mRNA or protein expression of VKOR (Fig. 7).

### Gegen *reduced the activity, as well as the mRNA and protein expression levels of TM in rat lung*

#### Inhibition of the TM activity

**Fig. 8:**
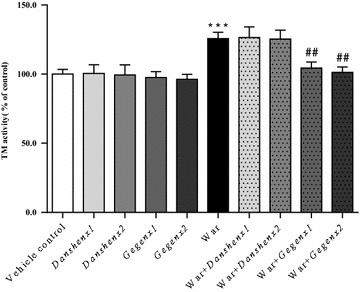
Fold of change in TM activity from different treatment groups. *P < 0.05; **P < 0.01; ***P < 0.001, compared with the vehicle control group. ^#^ *P* < 0.05; ^##^ *P* < 0.01; ^###^ *P* < 0.001, compared with the warfarin alone group. ^&^ *P* < 0.05; ^&&^ *P* < 0.01; ^&&&^ *P* < 0.001, compared within same groups of different doses. Each point represents the mean ± SD (n = 6)

Compared with the vehicle control group, there were no significant changes in the TM activities of the groups treated with *Danshen* or *Gegen*, although there did appear to be a decrease in the TM activities of the *Gegen*×1 and *Gegen*×2 groups. Warfarin led to a significant increase of 125.7 ± 4.2 % (*P* < 0.001) in the TM activity compared with the vehicle control group. A comparison of the warfarin-alone group with the combination groups (i.e., War + *Danshen*×1; War + *Danshen*×2; War + *Gegen*×1; War + *Gegen*×2) showed that *Danshen* did not affect the activity of TM during warfarin treatment. In contrast, the co-administrating of *Gegen* with warfarin led to significant decreases of 83.1 ± 3.2 % (*P* = 0.003) and 80.6 ± 2.9 % (*P* = 0.003) in the TM activities of the War + *Gegen*×1 and War + *Gegen*×2 groups, respectively. *Gegen* could therefore be used to offset the induction effect of warfarin towards TM activity (Fig. 8).

#### Down-regulation of the mRNA and protein expression levels of TM

**Fig. 9:**
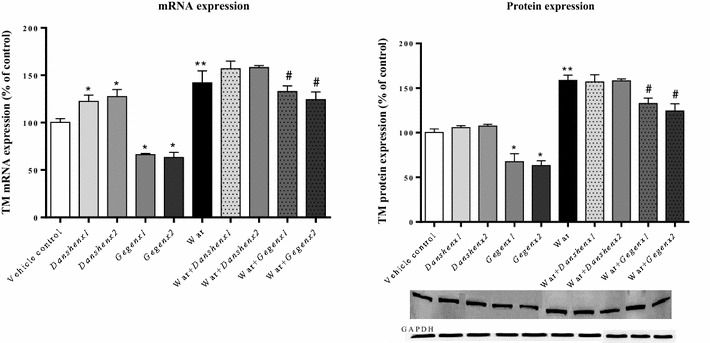
Fold of change in TM mRNA (*left*) and protein (*right*) expression from different treatment groups. ^*^ *P* < 0.05; ^**^ *P* < 0.01; ^***^ *P* < 0.001, compared with the vehicle control group. ^#^ *P* < 0.05; ^##^ *P* < 0.01; ^###^ *P* < 0.001, compared with the warfarin alone group. ^&^ *P* < 0.05; ^&&^ *P* < 0.01; ^&&&^ *P* < 0.001, compared within same groups of different doses. Each point represents the mean ± SD (n = 6)

Compared with the vehicle control group, no significant change was observed in the expression of the TM protein in the *Danshen* group, but we did observe an increase of almost 125 % (*P* = 0.02) in the mRNA expression of TM. In contrast, significant decreases of 66.3 ± 1.5 % (*P* = 0.03) and 63.3 ± 7.5 % (*P* = 0.03) were observed in the mRNA expression levels of TM in the *Gegen*×1 and *Gegen*×2 groups, respectively, compared with the vehicle control group. We also observed significant decreases of 67.6 ± 12.5 % (*P* = 0.03) and 63.3 ± 7.5 % (*P* = 0.03) in the expression levels of the TM protein in the *Gegen*×1 and *Gegen*×2 groups, respectively, compared with the vehicle control group. Significant increases of 141.9 ± 17.8 % (*P* = 0.003) and 158.6 ± 8.2 % (*P* = 0.002) were observed in the mRNA and protein expression levels of TM in the warfarin-alone group compared with the vehicle control group. Compared with the mRNA and protein expression levels of TM in the warfarin-alone group, we observed decreases of nearly 25 % (*P* = 0.03, mRNA expression) and 12 % (*P* = 0.04, protein expression), respectively, in the *Gegen* combination groups. Notably, the co-administration of *Danshen* with warfarin had no discernible impact on the mRNA or protein expression levels of TM compared with the warfarin-alone group (Fig. 9).

### Gegen led to a decrease in the plasma concentration of sTM

**Fig. 10:**
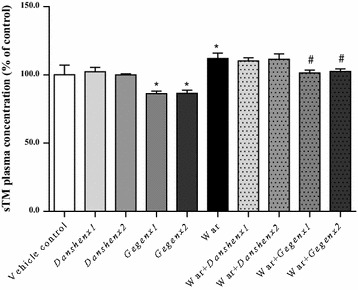
Fold of change in sTM plasma concentration from different treatment groups. ^*^ *P* < 0.05; ^**^ *P* < 0.01; ^***^ *P* < 0.001, compared with the vehicle control group. ^#^ *P* < 0.05; ^##^ *P* < 0.01; ^###^ *P* < 0.001, compared with the warfarin alone group. ^&^ *P* < 0.05; ^&&^ *P* < 0.01; ^&&&^ *P* < 0.001, compared within same groups of different doses. Each point represents the mean ± SD (n = 6)

Compared with the vehicle control group, there were no discernible changes in the plasma concentration of sTM in the *Danshen*×1 and *Danshen*×2 groups. In contrast, significant decreases of 86.3 ± 1.5 % (*P* = 0.01) and 86.5 ± 1.8 % (*P* = 0.01) were observed in the plasma concentrations of sTM in the *Gegen*×1 and *Gegen*×2 groups, respectively. Warfarin led to an increase in the plasma concentration of sTM to 112.0 ± 3.2 % (*P* = 0.03) compared with the vehicle control group. A comparison of the warfarin-alone group with the different combination groups (i.e., War + *Danshen*×1; War + *Danshen*×2; War + *Gegen*×1; War + *Gegen*×2) showed that *Danshen* had no discernible impact on the plasma concentration of sTM during warfarin treatment. In contrast, significant decreases of 11.4 ± 1.7 % (*P* = 0.03) and 10.2 ± 1.5 % (*P* = 0.03) were observed in the plasma concentrations of sTM for the War + *Gegen*×1 and War + *Gegen*×2 groups, respectively (Fig. 10).

## Discussion

*Danshen* and *Gegen* elicit their effects by improving microcirculation and inhibiting platelet aggregation [24, 32], whereas warfarin exerts its anticoagulant effect by inhibiting the activity of VKOR and inducing TM.

In the healthy Sprague–Dawley rats used in the current study, *Gegen* rather than *Danshen* interacted with warfarin to offset its anticoagulant effects. In the *Gegen*-warfarin interaction, *Gegen* could induce the phase I metabolism of warfarin in the liver by increasing the activities and mRNA expression levels of the different CYP isozymes, as well as inducing the activity and expression levels of VKOR and inhibiting those of TM. The results of our previous study showed that the administration of a DFG-warfarin combination led to a reduction in the warfarin plasma concentration and PT time of rats [23, 32]. Taken together with the results of the current study, it seems clear that these effects could be attributed to the *Gegen* present in DFG.

**Fig. 11:**
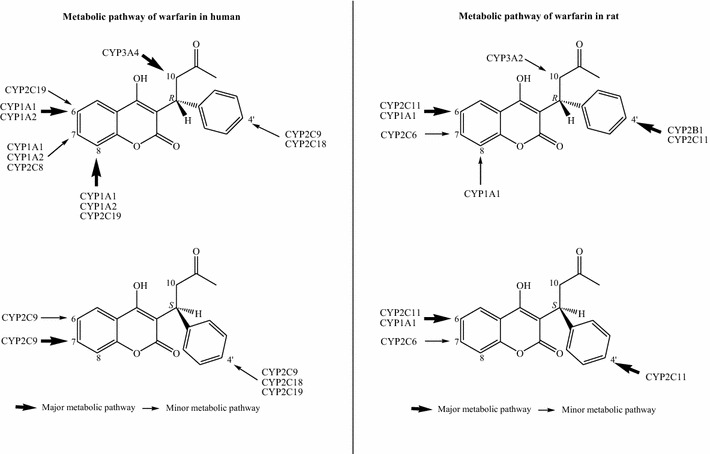
CYPs involved in warfarin metabolism in human and rat

Although there were no clinical reports in the literature pertaining to the interactions between *Gegen* and warfarin, the results of several case reports indicated that *Danshen* can interact with warfarin in humans [20–22]. However, these reports are inconsistent with our current findings. This discrepancy could be caused by several different factors, including (1) the use of different sources and preparations of *Danshen* (e.g., differences in the quality of the herbal medicines/contamination profiles); (2) a lack of adequate information in the documented case report (e.g., only one case report failed to infer a causal relationship); and (3) differences in the nature of the animals used in the different studies (e.g., rats versus humans). CYP enzymes represent some of the most highly conserved entities among different species, with relatively small differences in the primary amino acid sequences of the CYP enzymes across different species [50, 51]. Human CYP2C9, CYP2C19, CYP1A1 and CYP1A2 play important roles in warfarin metabolism, especially CYP2C9 [5], whereas the corresponding enzymes in rats are CYP2C11, CYP2C6, CYP1A1 and CYP2B1 (Fig. 11) [52]. Rat CYP1A1 shows a high level of conservation among species with greater than 80 % sequence identity compared with the corresponding enzyme in humans [50, 51]. The 6-, 7- and 8-mono-hydroxylated warfarin metabolites are formed by the CYP1A1 enzymes in rats and humans [50, 51]. Furthermore, gene sequence similarities of 74–80 % were reported for the CYP2C11 and CYP2C6 enzymes in rats compared with the human enzymes belonging to the CYP2C subfamily (mainly CYP2C9 and 2C19) [50, 51]. The 4′-, 6- and 7-mono-hydroxylated warfarin metabolites are mainly formed by CYP2C11 and CYP2C6 in rats. In humans, however, these metabolites are formed by CYP2C9, CYP2C19 and CYP2C18. Notably, a genetic similarity of 78 % was reported between the rat and human CYP2B1 enzymes, which are responsible for the formation of 4′-mono-hydroxylated warfarin [50, 51]. Based on the details provided above, CYP2C11, CYP2C6, CYP1A1 and CYP2B1 were selected in the current study to investigate warfarin-related interactions in a rat model. Although these isoforms are expressed in several different species, a high degree of similarity in their gene sequences between rats and human may not automatically result in similar levels of catalytic specificity and activity.

As well as assessing the impact of warfarin on the activities of CYPs, we also investigated the variability in the effects of warfarin in humans caused by polymorphisms in the CYP2C9 and VKORC1 genes [53]. Algorithms that integrate the relevant genetic and physical factors into comprehensive, individualized predictive models were used to predict warfarin dose [54]. Furthermore, physiological-based pharmacokinetic (PBPK) modeling and simulation software, including Simcyp, PK-Sim, GastroPlus and MATLAB Sim-Biology, were recently made available to quantitatively predict the drug/herb-drug interactions of warfarin in humans based on rat data [54].

The results of an in vitro study using HepG2 cells indicated that tanshinones could be used to induce the expression of CYP1A1 and CYP1A2 [55]. The treatment of rats with *Danshen*, which mainly consists of danshensu, SAB and tanshinones, led to an increase in the activity and protein expression of CYP1A2. However, the activity of CYP1A2 did no change when rat hepatocytes were treated with danshensu or SAB in isolation [56]. Furthermore, CYP1A-, CYP2C- and CYP3A-inducing agents were found in the ethyl acetate extract, but not in the aqueous extract of *Danshen* [57]. Taken together, the results of these previous studies indicate that the lipophilic tanshinones in *Danshen*, rather than the hydrophilic components, were responsible for inducing the activity and enhancing expression of the different CYP isozymes. We found that the major components in *Danshen* granules were hydrophilic compounds, including danshensu (1140.3 ± 24.6 µg/100 mg granules), SAB (821.3 ± 33.1 µg/100 mg granules) and PCA (82.2 ± 4.6 µg/100 mg granules), which might explain why *Danshen* showed no effect on the CYP activities or expression levels. This finding is also supported by Yueng’s study, where the major tanshinone components rather than the aqueous extract of *Danshen* showed effects on warfarin hydroxylation in vitro and in vivo [58].

Although paracetamol and sodium dehydroacetate (DHA-S) were reported to interact with warfarin by inhibiting VKOR activity, leading to hemorrhage in Sprague–Dawley rats, similar studies pertaining to the VKOR activity have never been conducted in context of herb-warfarin interactions [9, 10]. Regarding the results of previous studies on TM, the treatment of H9c2 cells with DFG led to a 5.36-fold up-regulation in the mRNA expression of TM compared with a control system [16]. Furthermore, SAB led to an 1.25- and 1.8-fold increases in the activity and mRNA expression of TM, respectively, over the control levels in human umbilical vein endothelial cells [15]. In our study, the mRNA expression of TM in the *Danshen*-treated group was slightly higher than that of the vehicle control group. However, there were no significant differences in the activity and protein expression levels of TM between these two groups. The inconsistencies observed between our own findings and those reported by other researchers could be attributed to differences in the nature of the experiments. For example, SAB was directly loaded onto the cells used in Shi’s study at a high concentration (0.0125–0.5 mg/mL) [15]. However, SAB was reported to be poorly absorbed in animal studies and has an extremely low systemic bioavailability [59]. In our study, although SAB was used as a major component of the *Danshen* granules, the in vivo effects were very different to those observed in cells because of the poor absorption and bioavailability of SAB. Moreover, the other components in the herbs could also act on the CYP, VKOR and TM enzymes. All of these factors could therefore be considered as potential explanations for the discrepancies observed between the in vitro and in vivo results.

## Conclusion

*Gegen*, rather than *Danshen* at the same tested dosage, offsets the anticoagulant effects of warfarin by accelerating the phase I liver metabolism of warfarin, as well as increasing the activity, mRNA and protein expression of VKOR while decreasing those of TM.

## Additional file

10.1186/s13020-016-0078-9 Animal liscence.

## Abbreviations

CYP: liver cytochrome P450
VKOR: vitamin K epoxide reductase
VKO: vitamin K_1_ 2, 3-epoxide
VK: vitamin K
TM: thrombomodulin
DGF: Danshen–Gegen Formula
SAB: salvianolic acid B
PB: phenobarbital
BNF: beta-naphthoflavone
CPA: cyclophosphamide
ELISA: enzyme-linked immunosorbent assay
