# Supplementary material for: Radix Puerariae lobatae (Gegen) suppresses the anticoagulation effect of warfarin: a pharmacokinetic and pharmacodynamics study
Source: Chin Med. 2016 Feb 27;11:7. doi: 10.1186/s13020-016-0078-9 (PMC4769563; doi:10.1186/s13020-016-0078-9)
Supplement: Supplementary file 1 — 10.1186/s13020-016-0078-9 Animal liscence. [file 13020_2016_78_MOESM1_ESM.pdf]

香港特別行政區政府
衞生署
香港灣仔皇后大道東 213 號
胡忠大廈 17 及 21 樓

THE GOVERNMENT OF THE HONG KONG
SPECIAL ADMINISTRATIVE REGION
DEPARTMENT OF HEALTH,
WU CHUNG HOUSE, 17TH & 21ST FLOORS,
213 QUEEN'S ROAD EAST, WAN CHAI,
HONG KONG.

本署檔號 OUR REF.: (14-960) in DH/HA&P/8/2/1 Pt.45

來函檔號 YOUR REF.:

電　話 TEL.: 2961 8931

圖文傳真 FAX.: 2127 7329

17 December 2014

GE Beikang
School of Pharmacy
The Chinese University of Hong Kong

Dear Sir/Madam,

Animals (Control of Experiments) Ordinance
Chapter 340

I refer to your application received on 18 November 2014 and forward herewith the following licence(s) issued under the Animals (Control of Experiments) Ordinance ("the Ordinance"):-

Form 2 : Licence to Conduct Experiments

Your attention is drawn to regulations 4 and 5 of the Animals (Control of Experiments) Regulations ("the Regulations") as excerpted below:-

"4. Records
Every licensee shall keep up-to-date a book in the form set out as Form 6 in the Schedule in which he shall record the particulars therein indicated of all experiments performed by him.

5. Returns
Every licensee shall render to the Director of Health on or before the 1st day of January each year a return in the form set out as Form 7 in the Schedule of all experiments performed by him during the preceding twelve months."

Copies of Form 6 and Form 7 are enclosed for your convenience. Failure to comply with section 11 and 12 of the Ordinance, and regulation 4 and regulation 5 of the Regulations is an offence and may result in prosecution and cancellation of your licence. Each offence would be punishable by a fine of HK$500[1] and to imprisonment for 3 months. You are reminded that your duty as a licensee under the said provisions subsists even if you subsequently cease to work or study in your academic institution/ organisation/ company.

[1] Note: Under section 113C(2) of the Criminal Procedure Ordinance, Cap. 221, where an Ordinance, provides for a fine, other than an excluded fine, for an offence expressed as an amount of money, the fine shall be deemed to be a fine at the level relevant to the amount of the fine in a prescribed table. For a $500 fine, the level applied should be Level 1 and thus, the fine could be up to $2000.

/P.2……

Please also be reminded that if you wish to continue your experiments after the specified periods as stated on the above licence / endorsement / teaching permit, you should renew them at least three months before the end-dates. You are reminded that it will be a breach of the Ordinance if you continue to conduct the experiments after the expiry date of the existing licence and before a new licence for the same experiments has been issued. On the other hand, if you have completed or stopped your experiments, or you are going to leave your academic institution/ organisation/ company before the specified periods, you should inform us in writing immediately and render the returns of experiments (Form 7) at the same time.

Yours faithfully,

(Dr Kellie SO)
for Director of Health

* *Remarks:-*

A "Code of Practice – Care and Use of Animals for Experimental Purposes" was prepared by the Agriculture, Fisheries and Conservation Department on the advice of the Animal Welfare Advisory Group.

Please visit the Agriculture, Fisheries and Conservation Department's website at <http://www.afcd.gov.hk/english/publications/publications_qua/files/code.pdf> for details of the Code of Practice.

<u>Encl.</u>

Form 2

Licence to Conduct Experiments

Name : GE Beikang [Ref No.: (14-960) in DH/HA&P/8/2/1 Pt.45]

Address : School of Pharmacy, The Chinese University of Hong Kong

By virtue of section 7 of the Animals (Control of Experiments) Ordinance, Chapter 340, the above-named is hereby licensed to conduct the type of experiment(s), at the place(s) and upon the conditions, hereinafter mentioned.

Type of experiment(s)

Rats will be used in the experiment. Dansheng/Gegen and warfarin will be administered orally to the animals. Jugular vein cannulation will be performed under anaesthesia. Multiple blood samples will be taken from the jugular vein. At the end of the experiment, the animals will be sacrificed by an overdose of anaesthetic. The condition of the animals will be monitored throughout the experiment. In case of serious injury or signs of severe distress/pain, the animals will be sacrificed.

Place(s) where experiment(s) may be conducted

Room 815, Lo Kwee-Seong Integrated Biomedical Sciences Building, The Chinese University of Hong Kong

---

Conditions

1. Such experiment(s) may only be conducted for the following purposes-

   To study the pharmacokinetics, pharmacodynamics and bioavailability of Dansheng/Gegen and warfarin using animals.

2. This licence is valid from 17 December 2014 to 16 December 2016

Dated 17 December 2014

DEPARTMENT OF HEALTH 衞生署 HONG KONG

Licensing Authority

ANIMALS (CONTROL OF EXPERIMENTS) REGULATIONS

FORM 6

Record of Experiments

Name of Licensee : ______________________

Date of experiment.	Place where performed.	Animal used.	Purpose of experiment.	If animal dead, state how died.	Nature of experiments (if performed under teaching permit or under special endorsement this should be stated).

ANIMALS (CONTROL OF EXPERIMENTS) REGULATIONS

FORM 7

Return of Experiments

Name of Licensee : ______________________

No. and date of Licence : ______________________

Period covered by return : From ____________ To ____________

Number of experiments conducted.	Place(s) where performed.	Kinds and number of animals used.	Purpose of experiments (e.g. manual skill, illustrating lectures, etc.).	Any teaching permits, lectures and other endorsements applicable during the period.

Dated ____________

Signed ______________________

(Licensee).

Annex – This section is not part of Form 7 but information supplied would facilitate our processing of your return.

Full name:* ______________________ *Contact No.:* ____________

HK Identity Card/Passport/ Travel Document No.: ____________ *Mobile No.:* ____________

Email Address: ____________ *(Facsimile)* ____________

Name of Institute/Company: ______________________

Name of Department: ______________________

*(Institute/Company chop)***

** Full name as appears on HK Identity Card/Passport/Travel Document*

*** Please obtain an official chop of the Institute/ Company where you are working or studying.*

Personal Information Collection Statement
Relating to Licence/ Permit/ Endorsement Issued under
the Animals (Control of Experiments) Ordinance, Chapter 340

Purpose of Collection

1. The personal data are provided by clients with whom the Department of Health (DH) interacts in the delivery of services, and other related activities. The personal data provided will be used by DH for the following purposes:

(a) processing applications for licences/ permits/ endorsements;
(b) recording purposes;
(c) statistical purposes; and
(d) for any other purposes permitted by law.

Failure to provide the requested personal data may lead to delay or an inability to process relevant licence/ permit/ endorsement application.

Classes of Transferees

2. The personal data you provided will be kept confidential for use within DH but they may also be disclosed to other Government bureaux/ departments or relevant parties for the purposes mentioned in paragraph 1 above, if required. Apart from this, the data may only be disclosed to parties where you have given consent to such disclosure or where such disclosure is allowed under the Personal Data (Privacy) Ordinance or any other legislation.

Access and Correction to Personal Data

3. You have the right of access and correction with respect to your personal data as provided for in Sections 18 and 22 and Principle 6 of Schedule 1 of the Personal Data (Privacy) Ordinance. Your right of access includes the right to obtain a copy of your personal data. A fee may be imposed for complying with a data access request.

Enquires

4. Enquiries concerning personal data provided, including the making of access and corrections, should be addressed to:

Principal Medical Officer (3)
Department of Health
Room 79, 21/F, Wu Chung House
213 Queen's Road East
Wan Chai, Hong Kong
Tel : 2961 8975

<u>Explanatory Notes to Returns of Experiments (Form 7) and its annex under the Animals (Control of Experiments) Ordinance (Cap. 340)</u>

- Pursuant to Regulation 5 of the Animals (Control of Experiments) Regulations (Cap. 340A), every licensee shall render to the Director of Health on or before the 1st day of January each year a return in the form set out as Form 7 in the Schedule of all experiments performed by him/her during the preceding twelve months.
- **<u>An annex to Form 7 has been added since 1 November 2013. The licensee shall provide his full name, HK identity card/ passport/ travel document number and contact information [name of institution/ company, name of department, contact phone number, mobile number, email address and facsimile number (if applicable)] while filling in the annex of the Form 7 for future correspondence if necessary.</u>**
- **<u>The licensee shall also obtain a department chop from an authorized person of the academic institution/ organisation/ company where he/ she is working or studying as an endorsement of the completed return form.</u>**
- For licensees performing the same experiment, every licensee is required to submit an individual return.
- Licensees holding more than one licence are required to submit a return for each licence.
- Licensees are advised to read the explanatory notes below before filling in the return form (Form 7):

1.	*No. and date of Licence.*	:	"No. of Licence" refers to the reference number, i.e. Ref No., stated on Form 2 (Licence to Conduct Experiments).<br>"Date of Licence" refers to the date on which the licence was issued.
2.	*Period covered by return.*	:	<u>Example 1:</u><br>If your licence was valid between 15 June 2012 and 14 June 2014, the period covered by return for the year 2013 is from 1.1.2013 to 31.12.2013.<br><br><u>Example 2:</u><br>If your licence was granted on 15 March 2013, the period covered by return for the year 2013 is from 15.3.2013 to 31.12.2013.<br><br><u>Example 3:</u><br>If your licence was valid until 15 September 2013, the period covered by return for the year 2013 is from 1.1.2013 to 15.9.2013.
3.	*Number of experiments conducted.*	:	Please state the exact number of experiments conducted within the period covered by return.

4.	*Place(s) where performed.*	: Please specify the exact location(s) of the place(s) where the experiments were conducted.
5.	*Kinds and number of animals used.*	: Please specify the types and exact number of animals used.
6.	*Purpose of experiments (e.g. manual skill, illustrating lectures, etc.).*	: Please state the purpose of the experiments.
7.	*Any teaching permits, lectures and other endorsements applicable during the period.*	: In addition to the Licence to Conduct Experiments (Form 2), please indicate whether you have been granted a Teaching Permit (Form 4) or Endorsement(s) (Form 3 and/or Form 5) for the experiment.
